# Development of a Cell Culture Chamber for Investigating the Therapeutic Effects of Electrical Stimulation on Neural Growth

**DOI:** 10.3390/biomedicines12020289

**Published:** 2024-01-26

**Authors:** Quy-Susan Huynh, R. M. Damian Holsinger

**Affiliations:** 1 Laboratory of Molecular Neuroscience and Dementia, School of Medical Sciences, Faculty of Medicine and Health, The University of Sydney, Camperdown, NSW 2050, Australia; qhuy6258@uni.sydney.edu.au; 2 Neuroscience, School of Medical Sciences, Faculty of Medicine and Health, The University of Sydney, Sydney, NSW 2006, Australia

**Keywords:** electrical stimulation, cell proliferation, cell migration, cell culture media, neural regeneration

## Abstract

Natural electric fields exist throughout the body during development and following injury, and, as such, EFs have the potential to be utilized to guide cell growth and regeneration. Electrical stimulation (ES) can also affect gene expression and other cellular behaviors, including cell migration and proliferation. To investigate the effects of electric fields on cells in vitro, a sterile chamber that delivers electrical stimuli is required. Here, we describe the construction of an ES chamber through the modification of an existing lid of a 6-well cell culture plate. Using human SH-SY5Y neuroblastoma cells, we tested the biocompatibility of materials, such as Araldite^®^, Tefgel™ and superglue, that were used to secure and maintain platinum electrodes to the cell culture plate lid, and we validated the electrical properties of the constructed ES chamber by calculating the comparable electrical conductivities of phosphate-buffered saline (PBS) and cell culture media from voltage and current measurements obtained from the ES chamber. Various electrical signals and durations of stimulation were tested on SH-SY5Y cells. Although none of the signals caused significant cell death, 3-(4,5-dimethylthiazol-2-yl)-2,5-diphenyltetrazolium bromide (MTT) assays revealed that shorter stimulation times and lower currents minimized negative effects. This design can be easily replicated and can be used to further investigate the therapeutic effects of electrical stimulation on neural cells.

## 1. Introduction

Electric fields (EFs) are naturally present in the human body and influence cellular activity. EFs with magnitudes of 10–75 mV/mm are present in the developing embryo and can influence morphogenesis [1]. These fields, however, shift over a lifetime [1] and, most notably, following injury [2]. For example, an EF of 25–40 mV/mm is formed between the apical and basolateral epithelial layers in the developing embryo [2]. When injury occurs, an EF develops lateral to the wound, and the injured site becomes more negatively charged [2]. Thus, the electrical current is driven in the direction of the wound that is considered to exert electrotactic effects on surrounding tissue [2]. The presence of these EFs appears to be important for healing and regeneration [2]. Reid and colleagues discovered that an eventual reversal in an EF during wound healing predicted the regeneration potential of tadpole tails [3]. Based on these concepts, electrically excitable tissues, such as muscle and neurons and the cells of the nervous system, have been targeted in the medical industry as potential treatment options following injury or neurodegeneration. Deep brain stimulation, the use of implanted brain electrodes to deliver pulses that excite neuronal tissue, is used to treat neurodegenerative diseases such as Parkinson’s disease [4], and electrodes of various designs are implanted to stimulate neural tissue to address types of hearing [5] and sight loss [6] or are used in bionic limbs [7].

Electrical stimulation (ES) is widely investigated on a cellular level to explore and elucidate the effects on electrotaxis [8,9], morphology [10], orientation [11], gene expression [12] and cell differentiation [13]. There is particular interest in the ES of cells in inherently electrically excitable tissues such as neuron and muscle, but fibroblasts [14], epithelial, osteoblasts [15] and stem cells [10,13,16] are also commonly used in studies. The outcomes of these studies vary, depending on the cell line, the electrical signal applied as well as the experimental apparatus employed.

Electrical signals can be categorized as direct current (DC) or alternating current (AC). In vitro studies utilize both DC [9,11] and AC [17,18] signals, but many implantable electronic devices such as cochlear implants use AC signals as these are considered biologically safe [19]. Signals can vary in current, voltage, frequency, waveforms and duty cycles [20].

In vitro studies have utilized various experimental devices to electrically stimulate cells. All these methods must deliver electrical signals to cells in a sterile environment and under physiological conditions. Direct coupling is the most basic system, involving two electrodes in direct contact with the cell culture media [21,22]. Due to the proximity of the electrodes within the cell culture media, less intense signals are utilized. There is also the possibility that redox by-products may form, that, in turn, induce changes in the pH of the media. To minimize the effect of these changes, salt bridges have been included in some designs. ES can also generate heat, which could be detrimental to cells and tissues. The effects of heat and redox by-products can be limited by employing lower currents, a shorter stimulation duration and the use of AC signals.

Capacitive stimulation is similar to direct coupling with the exception that the electrodes do not make physical contact with the cell culture media [23]. As the electrodes are not in direct contact with the media, stronger signals can be employed, but longer treatment durations are often required. This is similar to inductive systems that involve the generation of electromagnetic fields through the placement of conductive coils surrounding the cell culture system.

Metal electrodes, specifically those constructed from platinum, are commonly utilized in in vitro studies [22], as are those constructed from conductive polymers such as polypyrrole and blended materials incorporating nanoparticles or graphene [24,25]. These materials can be fabricated into scaffolds with manipulated surface topographies that mimic the extracellular matrix of cells [26,27]. These studies are significant, as combining the effects of scaffold topography with ES may be useful for tissue engineering applications [28].

Some studies have designed new ES chambers or bioreactors that completely replace the use of traditional cell culture plates [23], whilst others have modified existing plastic cell culture plates or petri dishes [21,22]. In the current study, we modified a Corning^®^ 6-well cell culture plate by attaching electrodes to the lid of the plate to develop an electrical stimulation chamber that could remain sterile and be utilized safely in a cell culture incubator.

## 2. Materials and Methods

### 2.1. Cell Culture

Human SH-SY5Y neuroblastoma cells (Cell Bank Australia, Westmead, NSW, Australia) were cultured in T25 flasks in complete culture medium consisting of 1:1 DMEM/HAMF12 (GIBCO, ThermoFisher Scientific, Scoresby, VIC, Australia), 10% fetal bovine serum, 1% sodium bicarbonate, 1% Penicillin-Streptomycin, 1% sodium pyruvate, 1% non-essential amino acids and 1% L-glutamine (all from GIBCO). The media were replaced every 2–3 days. When the flask was ~90% confluent, the flask was briefly rinsed in 1× PBS and 0.025% Trypsin/EDTA (GIBCO) was added to the flask and incubated for 3 min at 37 °C. Complete medium was added to the flask to neutralize the trypsin, and the cells were collected and placed in a 15 mL tube. The tube was centrifuged at 40 RCF for 3 min and the supernatant was removed and discarded. The cells were resuspended in fresh media and 1 × 10^4^ cells were added to each well of a 6-well plate. Media were changed after 3 days, and 3–4 days following seeding, the cells were electrically stimulated with various signals (details below) and maintained for 24 h following the end of stimulation before commencing the MTT assay.

Dose-dependent material toxicity was tested by initially seeding cells into a 24-well plate and growing them for 24 h. A 10 µL and 20 µL drop of undiluted material was placed into each well and incubated for 48 h. A third comparison involved placing the materials on a plastic pipette tip and dipping the pipette tip into the cell culture media for 2 s and removing it, ensuring no deposit was left behind.

To test for material cytotoxicity, cells were seeded in a 48-well plate. In a separate Eppendorf tube for each material, 5 µL of TefGel™, Araldite and Bostik superglue was added to 1 mL of distilled water and vortexed. From this mixture of water and material, 25 μL of solution was added to each well containing 225 µL of cell culture media for 0 min, 30 min, 3 h and 24 h before analysis via the MTT assay. Distilled water was used as the control. This test aimed to simulate material cytotoxicity if small amounts of material came into contact with the cells/media. It is important to note that the volume of media used is not be high enough to reach this section of the plate.

### 2.2. MTT Assay

Cell culture media were removed from the wells and replaced with serum-free DMEM/HAMF12 with 1% Penicillin-Streptomycin. MTT (Sigma-Aldrich, Macquarie Park, NSW, Australia) was dissolved in phosphate-buffered saline (PBS; GIBCO, Thermo Fisher Scientific, Scoresby, VIC, Australia) to a working solution of 5 mg/mL. The MTT solution was diluted to 0.5 mg/mL by adding the working solution to serum-free media. Following 2 h of incubation with MTT and the formation of purple formazan crystals in each well, the media were carefully removed and discarded. The formazan crystals in each well were dissolved using isopropanol containing 0.1% NP-40 and 4 mM HCl. The cell culture plate was covered in foil and placed on a rocker for 10 min. Three 100 µL aliquots were obtained from each well, placed into a 96 well-plate and analyzed using a CLARIOstar (BMG LABTECH, Ortenberg, Germany) spectrophotometer set at a wavelength of 560 nm.

### 2.3. Electrical Properties

An Economy Autorange Multimeter with a Non-Contact Voltage Sensor (Jaycar, Rydalmere, NSW, Australia; Cat No. QM1529) was used to determine the resistance between the solder and platinum (Pt) electrodes, solder and titanium (Ti) wire and Pt electrodes and Ti wires to ensure all values were below 5 Ω. The multimeter was placed in series to measure the current and in parallel for voltage. Various resistors were utilized to achieve specific current values.

**Figure 1:**
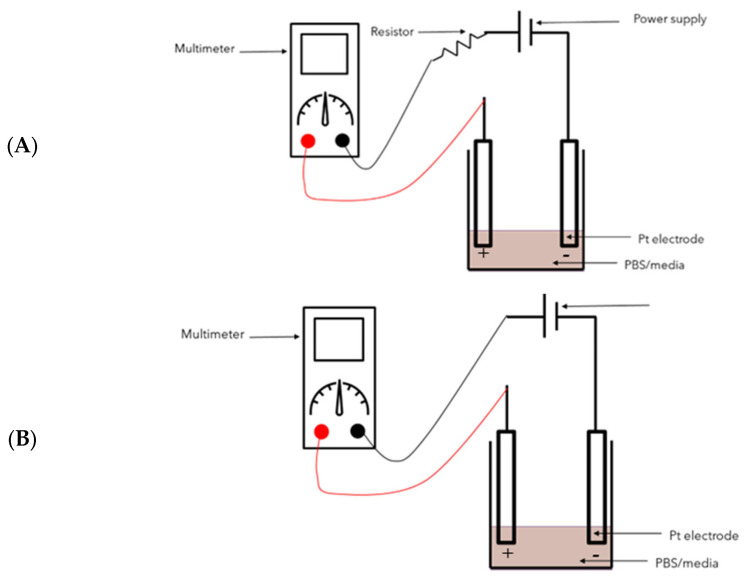
(**A**) Set-up used to verify the current and (**B**) set-up used to estimate the resistance of cell culture media and PBS.

To test the electrical properties of the plate, a resistor was placed in series to the 6-well plate. The multimeter was placed in series to measure the current (Figure 1A). The wells were connected in parallel. As the voltage was changed, resistors of varying values were used to establish a measured current of approximately 20–25 µA. Resistors used can be seen displayed on the abscissa (*x*-axis) of Figure 5a. A direct digital synthesis (DDS) signal generator/Counter (JDS6600; Walfront LLC, Lewes, DE, USA) was used to deliver biphasic pulses. The set voltage and resistance values used to generate the 20–25 µA of current were plotted, and linear regression was used to predict the current using the gradient of the curve according to Ohm’s law (V = IR). The average measured current and predicted current values obtained from linear regression were compared.

To approximate the resistance of the media, a multimeter was placed in series with the 6-well plate. No resistor was used (Figure 1B). The current was measured whilst adjusting the voltage. The current and voltage were plotted on a curve, and linear regression was used to predict the resistance by using the gradient according to Ohm’s law (V = IR).

### 2.4. Design of the ES Chamber

The ES chamber was designed to be conveniently connectable to an external signal generator whilst still remaining in cell culture incubators. The electrodes were constructed from conductive biocompatible materials that could be easily sterilised and remain sterile during situation.

Platinum (Pt) films on silicon discs were soldered to titanium (Ti) wires using Sn3.0Ag0.5Cu alloy solder. The 24-gauge Ti wire was shaped to the lip of the cell culture plate lid. The Ti wires were sufficiently thin to allow the cell culture plate to remain closed and maintain sterility. The resistance between the bottom of the electrode and titanium wires was measured to be less than 5 Ω. The electrodes were glued to the roof of the cell culture plate using Selleys Araldite (Selleys Australia, Padstow, NSW, Australia). Tefgel™ (Cannonvale, QLD, Australia) was used to coat the Araldite to prevent moisture from softening the material. Tefgel™ was also used to cover the solder to prevent moisture from oxidising the material. The Ti wires were employed to connect the electrodes to external function generators.

**Figure 2:**
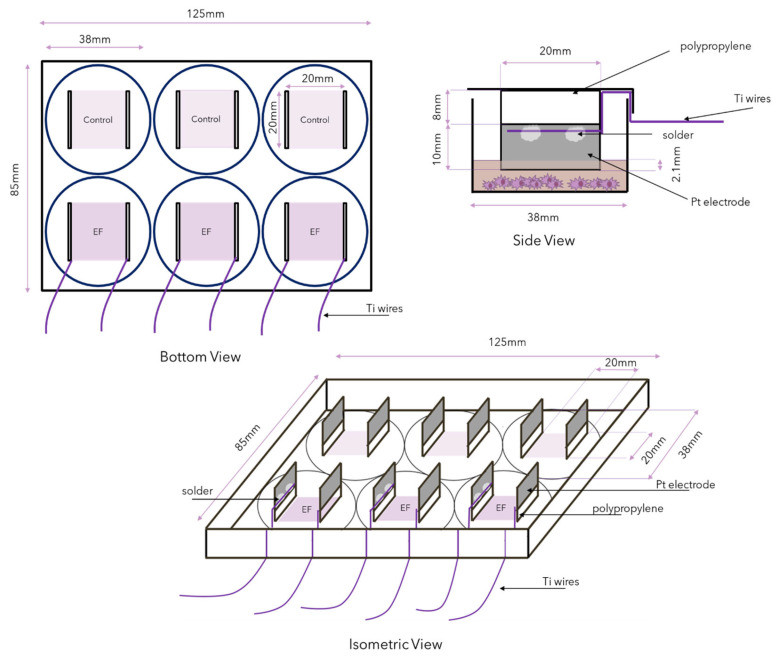
Bottom, side and cavalier oblique view of a modified lid of a cell culture plate. The electrodes are 20 mm in length and are placed 20 mm apart, creating a 20 × 20 mm EF. The Pt electrodes were soldered to Ti wires, which are sufficiently thin to pass through the lid of the culture plate without compromising sterility. Approximately 2.1 mm of the Pt electrode was submerged in the cell culture media but did not make contact with the bottom of the well when 2 mL of media was used.

The electrodes were spaced 20 mm apart, and the length of each electrode was 20 mm. Thus, an EF of 20 mm × 20 mm was created (Figure 2). To ensure that a substantial area of the electrodes was in contact with the media, without having to utilize large volumes of media, the electrodes were glued to 8 mm of polypropylene to extend the height of the electrodes to reach into the wells, without making contact with the bottom of the wells. The growth area of a well in a 6-well plate is approximately 9.6 cm^2^; hence, using 2 mL (2 cm^3^) of cell culture media resulted in approximately 2.1 mm of the Pt electrode being covered in media.

### 2.5. ES of Cells

Before ES, the spent media were removed and replenished with 2 mL of fresh media. The volume of 2 mL was important to cover a specified area of the platinum electrodes used to stimulate the cells. ES was performed inside the tissue culture incubator at 37 °C in 5% CO_2_. Platinum electrodes attached to the lid of the 6-well plate were connected to a circuit controlled by an Arduino nano (Jaycar Electronics, Rydalmere, NSW, Australia) for DC signals. An MTT assay was performed 24 h after stimulation.

## 3. Results

### 3.1. Cytotixicity of Materials

**Figure 3:**
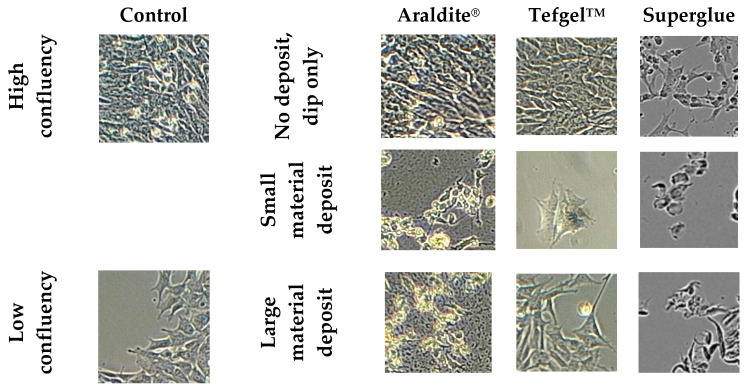
Brightfield images of SH-SY5Y cells 48 h following exposure to materials. Control column shows cell morphology in areas of high and low confluency for comparison to cells exposed to Araldite^®^, Tefgel™ and superglue. Short-term exposure and low amounts of Araldite^®^ are not toxic to cells. A 48 h exposure to small and large deposits led to cell death. Tefgel™ did not appear to have any adverse effect on cell morphology, irrespective of deposit size. Superglue appeared to be cytotoxic regardless of deposit size, even when the material was briefly dipped into cell culture media.

To determine the cytotoxicity of the materials used in the construction of the ES plate, we exposed human neuroblastoma SH-SY5Y cells to Araldite^®^, Tefgel™ and superglue for 48 h. Cell morphology was analyzed using light microscopic imaging (Figure 3).

Superglue appears to be the most toxic material, as even a brief dip of the material in the culture medium caused cell death (Figure 3). This was not evidenced for Tefgel^TM^ or Araldite^®^. Both superglue and Araldite^®^ displayed cytotoxic effects when small and large deposits were introduced to the culture media. Tefgel^TM^ did not appear to morphologically display any cytotoxic effects.

**Figure 4:**
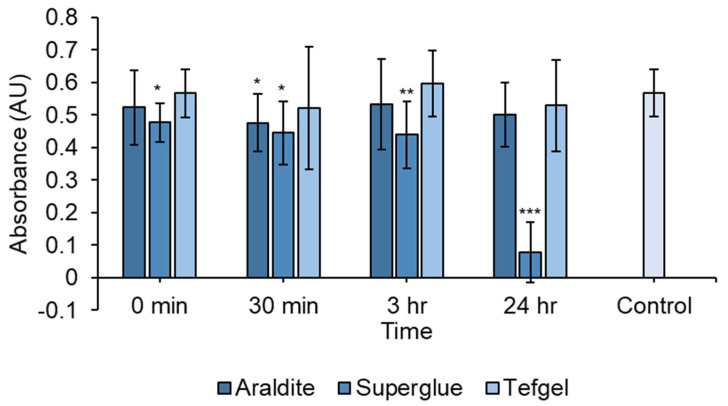
Cell viability evaluated using an MTT assay to determine the toxicity of potential materials to be used in the construction of the ES chamber. Materials were diluted in water and were at lower concentrations compared to those used in Figure 3. Cell viability was determined by comparing absorbance to distilled water (control). Tefgel™ did not appear to impart any adverse effects on cell growth. Additionally, Araldite^®^ does not appear cytotoxic at these lower concentrations. Conversely, superglue is cytotoxic even after dilution in water, and cells fail to recover following longer exposure. * *p* < 0.05, ** *p* < 0.01, *** *p* < 0.001 * indicate statistically significant difference to distilled water (control). Results shown as mean ± SD.

Material cytotoxicity was also determined by diluting the materials in distilled water and performing an MTT assay to evaluate cell viability (Figure 4). Once again, Tefgel™ did not appear to impart cytotoxic effects, whilst superglue remained cytotoxic, even at this lowered concentration. There was a statistically significant decrease for superglue at 0 min (*p* = 0.0133), 30 min (*p* = 0.0111), 3 h (*p* = 0.00977) and 24 h (*p* < 0.001) and Araldite^®^ at 30 min (*p* = 0.0355). Conversely, at this lowered concentration, Araldite^®^ did not appear to be as cytotoxic, as demonstrated in Figure 3, where solid Araldite^®^ was deposited into the well. As such, Araldite^®^ was selected as the material to glue components of the plate.

### 3.2. Verification of Electrical Properties of Stimulation Chamber

**Figure 5:**
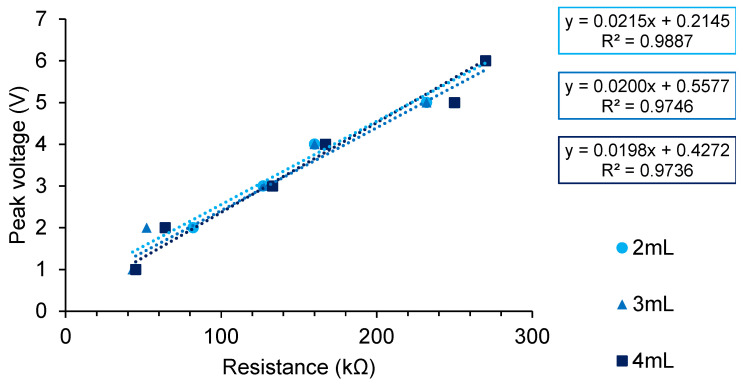
Relationship between set peak voltage and resistance from ES chamber using a square wave function at 2 Hz and 50% duty cycle. The set peak voltage and resistors used to produce measured currents in Table 1.

**Figure 6:**
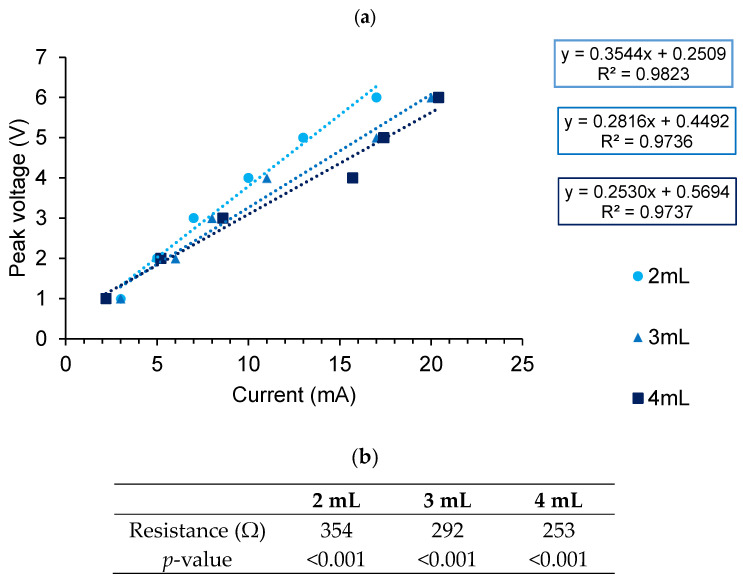
(**a**) Relationship between set peak voltage and measured current from ES chamber using square wave function at 2 Hz 50% duty cycle. No resistors were used, and Ohm’s law (V = IR) was used to infer resistance of media from the gradient using different volumes of media. Figure 1b depicts the set-up used in to measure the current. Resistance appears to decrease with increased volumes of media. (**b**) Table depicting resistance values and *p*-values of linear regression for data in (**a**).

The stimulation chamber follows the rules of Ohm’s law when used with either phosphate-buffered saline (PBS) or DMEM/F12 media. Comparison between different media compositions used for different cell lines did not affect the results (Figure 5 and Figure 6 and Table 1), evidenced by the use of the same resistor values for similar stimulation parameters. Additionally, PBS did not appear to differ substantially in conductivity to cell culture media.

Comparison of average maximum current measured in Table 1 to predicted current from linear regression show high percentages of error. Figure 1A depicts the set-up used in order to measure the current.

### 3.3. Validation Experiments

As a means of validating our 6-well ES design, we compared our results to published values in the literature [29,30,31]. Resistance (R) is the measure of opposition to electron flow. A high resistance suggests high opposition to electron flow. Resistance depends on the resistivity (ρ) of the material as well as the length (L) and cross-sectional area (A) of the material used (Equation (1)).

(1) $$Resistance\;(\Omega )=resistivity\;(\Omega \cdot m)\;\frac{length\;(m)}{area\;(m^{2})}=\frac{voltage\;(V)}{current\;(A)}\;R\;\left(\Omega \right)=\rho \;\left(\Omega \cdot m\right)\frac{L\;\left(m\right)}{A\;\left(m^{2}\right)}=\frac{V\;(V)}{I\;(A)}$$

**Equation (1)**: Resistance (R) is related to the resistivity (ρ), length (L) and cross-sectional area (A) of the material used in stimulation. Resistance can also be determined using Ohm’s law and found by dividing the measured voltage (V) measured in volts by the measured current (I) in amperes.

Unlike resistance, resistivity (ρ) is dependent on the characteristics of the material and indicates whether the material, regardless of dimensions, has high or low resistance to electron flow. Electrical conductivity is the inverse of resistivity (Equation (2)) and, hence, indicates how well a material allows electron flow, regardless of dimensions.

(2) $$Resistivity\;\left(\Omega \cdot m\right)=\frac{1}{electrical\;conductivity\;(Sm^{-1})}\;\rho \;\left(\Omega \cdot m\right)=\frac{1}{\sigma (Sm^{-1})}$$

**Equation (2)**: Resistivity (ρ) is the inverse of electrical conductivity (σ) as resistivity is a measure of opposition to electron flow due to material properties. Resistivity and electrical conductivity are independent of material dimensions.

**Figure 7:**
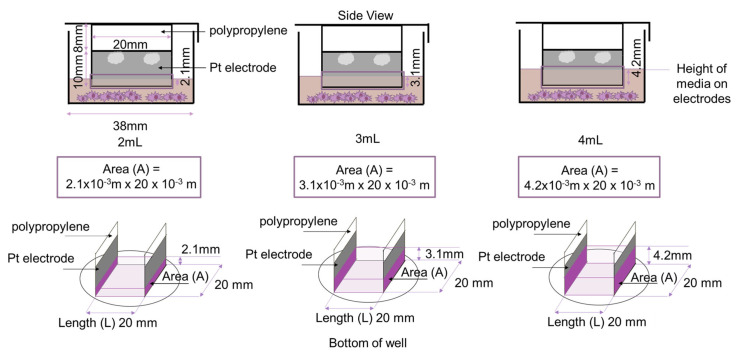
Diagram depicting the area (A) (marked in dark purple) and length (L) used to estimate resistance values of electrical conductivity of PBS and cell culture media from published data [29,30,31]. The value of A changes as a result of increased volume of media, which increases the area of the electrodes covered by liquid.

We used electrical conductivities (σ) obtained from published data [29,30,31] to calculate and compare resistance. The electrical conductivity of fresh media was estimated to be 1.68 Sm^−1^ [29], and commercial 1× PBS was between 1.4 and 2 Sm^−1^ [30,31]. The results are shown in Table 2 and Table 3 and Figure 7.

The calculated values obtained from published data (Table 3) are similar in magnitude to our results (Figure 6b). Based on the percentage errors, our results suggest that the electrical conductivity of the PBS used is closer to 1.4 Sm^−1^ [30,31] (Table 2). Hence, our resistance values match published data, thus validating and verifying the electrical properties of our constructed ES chamber.

### 3.4. Observations

**Figure 8:**
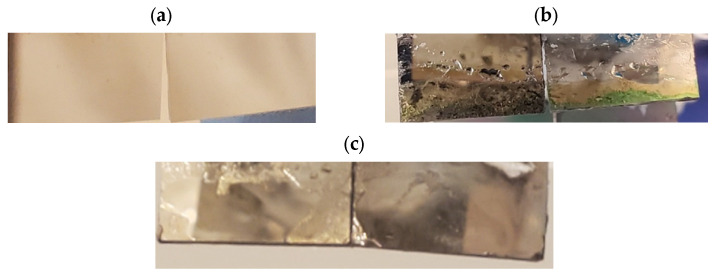
(**a**) New Pt electrodes. (**b**) Corroded Pt electrodes from 3 V 80–90 µA stimulation. (**c**) Pt electrodes following several cycles (in excess of 200 h) of stimulation. (**b**,**c**) are images taken from the positive electrode. Both positive and negative electrodes look similar.

Although platinum is considered an inert electrode, the metal can still undergo redox reactions [32]. Low currents were utilized, not only for cytotoxic reasons but also for the longevity of the platinum electrodes. We first tested the limits of the parameters that could be used on cells by applying a stimulus of 3 V with a current of 80–90 µA for 3 h. Following the conclusion of this trial, we observed that all the cells in the well had detached and eventually died. We also observed that the platinum film had corroded and that the silicon base under the platinum had become exposed (Figure 8b). Corrosion was not observed on electrodes that were used for over 200 h (Figure 8c).

### 3.5. Cell Viability of ES Using 2 V DC EF on SH-SY5Y Cells

**Figure 9:**
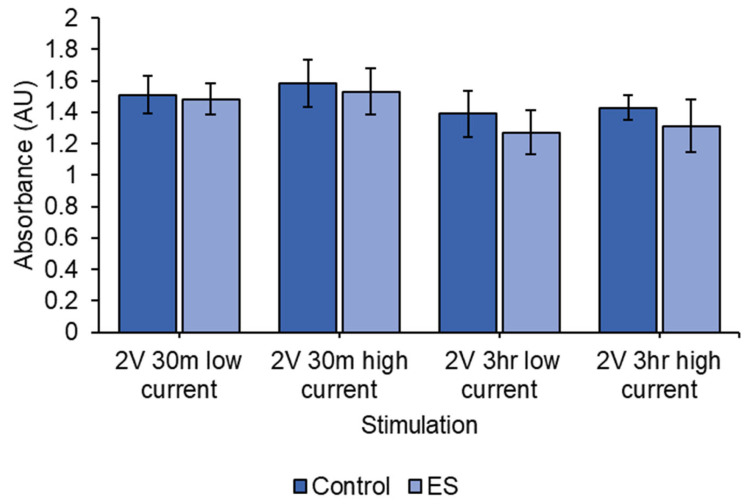
MTT assay results for 2 V DC-treated cells. Current was varied between a low current (0.3–0.4 µA) and high current (20–28 µA), and the length of treatment was varied between short (30 min) and long (3 h) stimulation time. Low currents and shorter stimulation times appear to limit cell death. No significant differences between control and ES cells were observed. 2 V DC 3 h high current (*p* = 0.233), 2 V DC 3 h low current (*p* = 0.258), 2 V DC 30 min high current (*p* = 0.613) and 2 V DC 30 min low current (*p* = 0.754). Results shown as mean ± SD.

There were no statistical differences between control and ES for any of the stimulations in Figure 9 (2 V DC 3 h high current (*p* = 0.233), 2 V DC 3 h low current (*p* = 0.258), 2 V DC 30 min high current (*p* = 0.613) and 2 V DC 30 min low current (*p* = 0.754)). Lower currents and shorter stimulation times improved cell viability.

## 4. Discussion

The application of small electrical currents to aid wound healing and nerve regeneration is a rapidly expanding field of research. The testing of parameters in vitro requires the design and construction of electrical stimulation (ES) chambers or bioreactors. Here, we describe the successful development and construction of a Corning^®^ 6-well cell culture plate lid that was then used repeatedly, as an ES chamber. The durability of this ES chamber was demonstrated by the ability to repeatedly sterilize and re-use the lid for a period in excess of one year. The ES chamber was able to provide electrical signals to the cells through conductive platinum electrodes, as seen in Figure 6. The material and plate were easily sterilized, as evidenced by an absence of contamination in any of the experiments. The design is attached to the lid of the cell culture plate and is easily transferrable across multiple 6-well cell culture plates. The lid is easily constructed and replicable should new lids need to be made. The design does not affect how a standard cell culture plate functions. When constructing new electrical stimulation chambers from materials other than polystyrene [23], additional factors, such as leak prevention, maintaining a sterile environment and cell attachment, must be considered. These factors are not an issue when modifying a standard cell culture plate.

We evaluated different adhesive materials to construct the ES chamber. Tef-gel^TM^ was considered the safest as it did not cause cell death since the material does not contain solvents and consists of polytetrafluoroethylene (PTFE) [33]. This polymer is utilized in tissue engineering applications [34] and, hence, was deemed safe to use to protect the soldering components from corrosion. Superglue is a cyanoacrylate-based material that has been utilized for biomedical applications [35]. Landegren and colleagues reported that, although cyanoacrylate-based materials displayed initial cytotoxic effects, cells were able to recover from this cytotoxicity following several days of culture in the presence of the compound [36]. At 24 h following exposure to superglue, Landegren et al. observed cell death and reduced cell size with rounded cell morphology [36]. These results were similar to our findings (Figure 4) and suggest that despite initial mild toxicity, the cells adapt to their environment, probably due to the dilution of the mildly toxic chemicals over a period of hours.

We utilized the modified cell culture plate to estimate the resistance of PBS and cell culture media (Table 2 and Table 3). Lang et al. used a conductivity probe to estimate the electrical conductivity (σ) of DMEM media containing 10% FBS with 1% streptomycin [29]. The electrical conductivity of fresh media was estimated to be 1.68 Sm^−1^, and this value varied between 1.728 and 1.976 Sm^−1^ over time, as cells were cultured in the media for 24–72 h [29]. The change in electrical conductivity was due to changes in salt concentrations that occurred as the cells grew in the culture medium [29]. Lang and colleagues similarly found that different media compositions with the same FBS concentration had similar electrical conductivities [29]. When the FBS concentration was increased, electrical conductivity decreased as a result of lower salt concentrations [29]. The electrical conductivity of commercial 1× PBS lies between 1.4 and 2 Sm^−1^ [30,31]. This also confirms why there appeared to be very little difference between cell culture media and PBS solutions (Figure 5 and Figure 6). The electrical resistance values obtained in our experiments were comparable to those observed by Lang and colleagues [29] and published data on PBS [30,31] (Table 2, Table 3 and Table 4).

To validate our results, we used the electrical conductivity of cell culture media that Lang and colleagues obtained as well as published values of electrical conductivity of PBS to calculate theoretical resistance values (Table 2 and Table 3), and they then compared them to the resistance values obtained in our experiments (Table 4). The values obtained are similar in magnitude to our results but vary due to different methods of measurement. Based on the percentage errors, our results suggest that the electrical conductivity of PBS is closer to 1.4 Sm^−1^ [30,31] (Table 2). The decreased resistance of the media as a function of volume can be explained using Equation (1) ($R=\rho \;\frac{L}{A}$), where resistivity (ρ) and length (L) are constants, and, therefore, resistance (R) is inversely proportional to area (A).

A limitation of the ES plate is the corrosion of Pt electrodes, which eventually need to be replaced. To prevent the build-up of redox by-products, the plate could be improved through the addition of a salt bridge, but this would increase the complexity of the chamber and also introduce sterility issues [32,36]. The ES chamber designed here is best used for shorter stimulation periods and lower currents that would improve the longevity of the plate and limit cell death through the build-up of redox products [21,37]. As seen in Figure 8c, short stimulation durations and low currents protected the electrodes when used for over a year compared to when longer duration (3 h) and high currents (150 mV/mm DC EF generating currents of 80–90 µA) were employed (leading to corrosion of the Pt electrodes; Figure 8b). Hence, to increase the longevity of the modified cell culture ES chamber, low currents and short stimulation times are recommended.

In this series of experiments, we discovered that that lower currents and stimulation periods generated the best outcome on the survival of SH-SY5Y cells (Figure 9). A study by Ghasemi-Mobarakeh and colleagues, who employed an electrically conductive polymer to stimulate cells at 1.5 V for one hour, discovered that these parameters were suitable to stimulate cell proliferation [38]. They observed an increased difference between stimulated and non-stimulated cells over a period of 1, 3 and 5 days. These differing results could be attributed to the different ES parameters and the different lengths of the study. Koppes and colleagues found that increasing the EF strength to 200 mV/mm with 1 mA currents resulted in abnormal Schwann cell morphology [39]. In a study employing HeLa cells, Ganeson and co-workers found that increasing the EF strength and pulse width led to decreased cell viability, although these investigators suggested that their observations may have been caused by the electroporation parameters that were employed [40]. Compared to the investigations mentioned above, we utilized parameters that were in the low micro ampere range. However, Lyte and colleagues also used currents between 10 and 24 μA and voltages between 30 and 50 V to stimulate EL4 lymphoma cells for a period of 20–24 h, but they reported a noticeable decline in cell proliferation [41]. A difference between the current study and that of Lyte and colleagues was the cell line that was employed, which may imply that electrically excitable cells such as neurons and muscle cells may be more suited to these studies. An in vivo study utilizing the cochlea from guinea pigs found that spiral ganglion density was increased following a chronic, 9-week ES [42]. A pairwise comparison between stimulated and non-stimulated cochleas revealed that ES at currents between 100 and 400 µA improved spiral ganglion density. These studies demonstrate the importance of investigations such as ours that aim to improve the practicality and feasibility of electrical stimulation chambers for studying the effects of electrical currents in wound healing and neuron regeneration.

## 5. Conclusions

We designed a robust electrical stimulation (ES) chamber that fulfills important requirements for use in cell culture experiments. The modified chamber, built based on a 6-well tissue culture plate format, can withstand repeated sterilization and incubator conditions and was produced with materials that displayed minimal cytotoxic effects. Our ES chamber can be utilized to study the effects of ES on cells and is best for investigating low currents and short stimulation periods. These ES parameters can be used to study the potential therapeutic effects of ES, such as increasing the cell proliferation, migration and differentiation of stem cells down a specific lineage, increasing gene expression and the secretion of trophic factors. The chamber is durable and was used repeatedly for over 12 months, with no loss of conductivity or resistance.

## Figures and Tables

**Table 1:** The gradient in Figure 5 represents the current as defined by Ohm’s law; V = IR. Linear regression was used to determine the gradient of the curve and predict a current value for varying volumes of media. Linear regression analysis was significant (*p* < 0.001) for all gradients.

Peak Voltage (V)	Measured Current (µA) 2 mL	Measured Current (µA) 3 mL	Measured Current (µA) 4 mL
1	25	24	23
2	24	25	24
3	26	24	26
4	22	26	25
5	22	22	26
6	23	23	24
Average	23.7	24.0	24.7
Predicted	21.5	20.0	19.8
*p*-value for gradient	<0.001	<0.001	<0.001
%error	10.23%	20.00%	24.75%

**Table 2:** Electrical conductivity values from published data [29,30,31].

Electrical Conductivity (*σ*) (Sm^−1^)	Calculation of Resistivity	Resistivity (*ρ*) (Ω · m)	Reference
Fresh media—1.68	$\rho =\frac{\;1}{\sigma }=\;\frac{1}{1.68\;Sm^{-1}}$	0.595	[29]
PBS—1.4	$\rho =\frac{\;1}{\sigma }=\;\frac{1}{1.4\;Sm^{-1}}$	0.71	[30,31]
PBS—2.0	$\rho =\frac{\;1}{\sigma }=\;\frac{1}{2.0\;Sm^{-1}}$	0.50	[30,31]

**Table 3:** Values and equations used to determine the resistance of the media and PBS from electrical conductivity from published data [29,30,31] in order to compare to our data. Resistivity values were obtained from Table 2 and A values were from Figure 7.

Resistivity (ρ) (Ω · m)	Area (A) (m^2^)	Volume of Media (mL)	Calculation
0.595	4.2 × 10^−5^	2	$R=0.595\;\Omega \cdot m\;\frac{20\;\times \;10^{-3}m}{4.2\;\times \;10^{-5}m^{2}\;}$
	6.2 × 10^−5^	3	$R=0.595\;\Omega \cdot m\;\frac{20\;\times \;10^{-3}m}{6.2\;\times \;10^{-5}m^{2}\;}$
	8.4 × 10^−5^	4	$R=0.595\;\Omega \cdot m\;\frac{20\;\times \;10^{-3}m}{8.4\;\times \;10^{-5}m^{2}\;}$
0.71	4.2 × 10^−5^	2	$R=0.71\;\Omega \cdot m\;\frac{20\;\times \;10^{-3}m}{4.2\;\times \;10^{-5}m^{2}\;}$
	6.2 × 10^−5^	3	$R=0.71\;\Omega \cdot m\;\frac{20\;\times \;10^{-3}m}{6.2\;\times \;10^{-5}m^{2}\;}$
	8.4 × 10^−5^	4	$R=0.71\;\Omega \cdot m\;\frac{20\;\times \;10^{-3}m}{8.4\;\times \;10^{-5}m^{2}\;}$
0.50	4.2 × 10^−5^	2	$R=0.50\;\Omega \cdot m\;\frac{20\;\times \;10^{-3}m}{4.2\;\times \;10^{-5}m^{2}\;}$
	6.2 × 10^−5^	3	$R=0.50\;\Omega \cdot m\;\frac{20\;\times \;10^{-3}m}{6.2\;\times \;10^{-5}m^{2}\;}$
	8.4 × 10^−5^	4	$R=0.50\;\Omega \cdot m\;\frac{20\;\times \;10^{-3}m}{8.4\;\times \;10^{-5}m^{2}\;}$

**Table 4:** Calculated values of resistance using data in Table 2 and comparing to our experiment (bold text). Percentage error was also calculated to compare results. Media and PBS used in our experiments appear to have electrical conductivities closest to 1.4 Sm^−1^.

	2 mL	3 mL	4 mL
Resistance (R) (Ω)	354	292	253
R calculated from [29] (Ω)	283	190	142
% error	25.1	53.7	78.2
R from PBS (1.4 Sm^−1^) (Ω)	340	230	170
% error	4.08	26.7	48.8
R from PBS (2 Sm^−1^) (Ω)	238	161	119
% error	48.7	81.0	113

## Data Availability

Data are contained within the article.
